# Short sleep, lifestyle, cardiovascular risk, and health awareness among Polish final-year secondary school students: findings from a nationwide study

**DOI:** 10.3389/frsle.2026.1866589

**Published:** 2026-07-24

**Authors:** Dorota Godzina, Agnieszka Doryńska, Ludmiła Podgórska-Stawiecka, Anna Mizerska, Witold Smigielski, Piotr Dobrowolski, Aleksander Prejbisz

**Affiliations:** Center for Cardiovascular Disease Prevention and Epidemiology, National Institute of Cardiology, Warsaw, Poland

**Keywords:** cardiovascular risk, health awareness, lifestyle behaviors, short sleep, sleep duration, young adults (18–25 years old)

## Abstract

**Background:**

Sleep is an important regulator of metabolic and cardiovascular health. Insufficient sleep is associated with adverse health behaviors and increased cardiovascular risk in young adults. Understanding sleep patterns and risk awareness in this group is essential for prevention.

**Methods:**

A nationally representative cross-sectional survey was conducted among 9,874 final-year Polish secondary school students. The questionnaire assessed sleep duration, physical activity, diet, alcohol and tobacco use, screen time, body mass index (BMI) and blood pressure. Associations between weekday sleep duration and lifestyle factors and cardiovascular risk were analyzed.

**Results:**

Over 46% of students reported sleeping less than 7 h on weekdays, with longer sleep on weekends (6.7 ± 1.3 vs. 9.0 ± 1.5 h, *p* < 0.001). Short sleep was associated with higher consumption of processed foods, alcohol use, tobacco smoking, lower winter physical activity, and greater screen time. Students sleeping <7 h were more often underweight or obese. Awareness of short sleep as a cardiometabolic risk factor was limited.

**Conclusions:**

Insufficient sleep is common among Polish young adults and is associated with an unfavorable behavioral and cardiometabolic risk profile.

## Introduction

Sleep is one of the basic physiological processes that determine the proper functioning of the body. In recent years, increasing attention has been paid to the role of sleep in the pathogenesis of cardiovascular disease (CVD) (Grandner et al., 2016; Nautiyal et al., 2024). Short sleep duration is increasingly recognized as a modifiable risk factor for cardiometabolic health in young adults (Itani et al., 2017; St-Onge et al., 2016; Chaput et al., 2018). Evidence from systematic reviews, meta-analyses, and expert consensus statements indicates that insufficient sleep is associated with obesity, hypertension, impaired glucose metabolism, and an increased risk of cardiovascular and metabolic disorders (Itani et al., 2017; St-Onge et al., 2016; Chaput et al., 2018).

A meta-analysis conducted by Hosseini et al., showed that chronic sleep deprivation (< 6 h) is associated with an increased risk of developing hypertension (Hosseini et al., 2024). In turn, the European Society of Cardiology (ESC) in its 2021 guidelines on primary prevention of CVD recognized sleep disorders—both sleep deprivation and poor sleep quality—as an independent risk factor for CVD, emphasizing the need to include sleep in population prevention strategies (Visseren et al., 2021). Short sleep not only directly affects cardiovascular parameters, but also correlates closely with other unhealthy behaviors. Studies have shown that individuals who sleep less tend to consume more calories, follow poorer-quality diets (Dashti et al., 2015), are less physically active (Kim et al., 2016), use tobacco and alcohol more frequently (Liu et al., 2025), and spend more time in front of screens (Kim et al., 2016). The coexistence of these lifestyle factors promotes the development of obesity, diabetes, and inflammation, which are strongly associated with the development of atherosclerosis and its complications (Dobrowolski et al., 2022). However, despite growing evidence regarding these adverse health consequences, little is known about young people's awareness of sleep-related cardiometabolic risks.

To address this knowledge gap, we conducted a nationally representative survey of nearly 10,000 final-year Polish secondary school students who are at the threshold of adulthood, a critical period for establishing long-term health behaviors and lifestyle patterns. In this large-scale study, we comprehensively assessed the relationships between sleep duration, lifestyle factors, cardiovascular risk factors, and health-related behaviors, including physical activity, dietary habits, alcohol consumption, tobacco use, screen exposure, and knowledge of cardiovascular disease. More than 46% of participants reported sleeping less than 7 h on weekdays, and short sleep duration was associated with a range of less healthy behaviors. Furthermore, our findings indicate that awareness of sleep as a cardiovascular and metabolic risk factor remains limited among young people.

## Methodology

The study was conducted as part of a nationwide survey project involving a random, and representative sample of final-year secondary school students in Poland. The main results of the study and its methodology are described in detail (Dobrowolski et al., 2026). Data were collected from 10095 students from 227 schools (high schools and technical schools) between October 2023 and January 2024. The present article analyzes data from 9,874 participants who answered questions about sleep duration and screen time.

### Selection of schools and participants

The list of schools was obtained from the publicly available database of the Ministry of Education and Science (https://dane.gov.pl). From this list, 250 schools were randomly selected, maintaining the proportion of the population of final-year secondary school students in each province, based on data from the Central Statistical Office. The schools were selected to represent both provincial capitals and smaller towns. Ultimately, 227 schools agreed to participate, resulting in a high response rate of 90.8%. In each school, students in the final grades were selected at random or in alphabetical order.

### Characteristics of the survey

The survey was completely anonymous and, in accordance with Polish law, did not require the approval of a bioethics committee. Its aim was to assess the knowledge and lifestyle of young people in the context of cardiovascular disease risk.

The questionnaire consisted of 20 questions. The first three concerned demographic data—gender, height, and weight—and a subjective assessment of one's own body shape. Based on the declared height and weight, the BMI was calculated and classified according to the WHO definition: < 18.5 kg/m^2^—underweight; 18.5–24.9 kg/m^2^—normal weight, 25.0–29.9 kg/m^2^—overweight, and ≥30.0 kg/m^2^—obese.

Additional questions concerned the parents‘ level of education and the participants' history of blood pressure measurements. Those who knew their results were asked to provide them. Hypertension was defined as systolic blood pressure ≥140 mm Hg or diastolic blood pressure ≥90 mm Hg. Blood pressure data were self-reported and were not verified by direct measurements performed within the study.

The subsequent sections of the questionnaire assessed physical activity, distinguishing between summer and winter periods. Participants who reported moderate physical activity for more than 150 min per week or vigorous physical activity for at least 75 min per week in accordance with WHO guidelines, or an equivalent combination of moderate- and vigorous-intensity physical activity throughout the week, were considered to meet the recommendations.

Participants were also asked about the time spent in front of screens (TV, tablet, phone, computer) and the number of hours of sleep on weekdays and weekends. Sleep duration of less than 7 h per day was classified as insufficient. The study also assessed respondents' awareness of the impact of sleep deprivation on the risk of cardiovascular disease. Sleep duration was assessed using self-reported questionnaire data only. The survey did not include validated instruments assessing other dimensions of sleep health, such as sleep quality, insomnia symptoms, sleep regularity, daytime sleepiness, sleep-disordered breathing, or other sleep-related disorders.

The section on alcohol analyzed consumption patterns, using a threshold of ≥100 g of pure alcohol per week (equivalent to six large beers, one bottle of wine, or 300 ml of vodka) as excessive consumption.

The section on smoking distinguished between the use of classic tobacco products (cigarettes, cigars, pipes) and alternative products (e-cigarettes, smokeless products, herbal smoking products, and THC). Respondents indicated whether they considered particular foods to be beneficial or harmful to health, especially in the context of the risk of heart attack or stroke, and indicated which of these they had consumed in the past week.

The last section of the questionnaire concerned general knowledge about cardiovascular diseases, including their role as the leading cause of death in Poland and awareness of the most important risk factors for myocardial infarction.

### Statistical analysis

The distributions of numerical variables were assessed using the Shapiro-Wilk test. For continuous data, means ± standard deviation or medians with interquartile range were used. Qualitative variables were presented as absolute numbers and percentages.

For group comparisons, the Student's t-test or Mann-Whitney U test was used for quantitative variables, and the chi-square test with Yates correction was used for qualitative variables. All results were weighted by gender, province, and school location (provincial capital vs. other). The analyses were performed using Stata 18.0 (StataCorp, USA), with a statistical significance level of *p* < 0.05.

## Results

Responses from 9874 students in the final years of secondary school were analyzed. Women accounted for 49% of the total sample.

**Figure 1:**
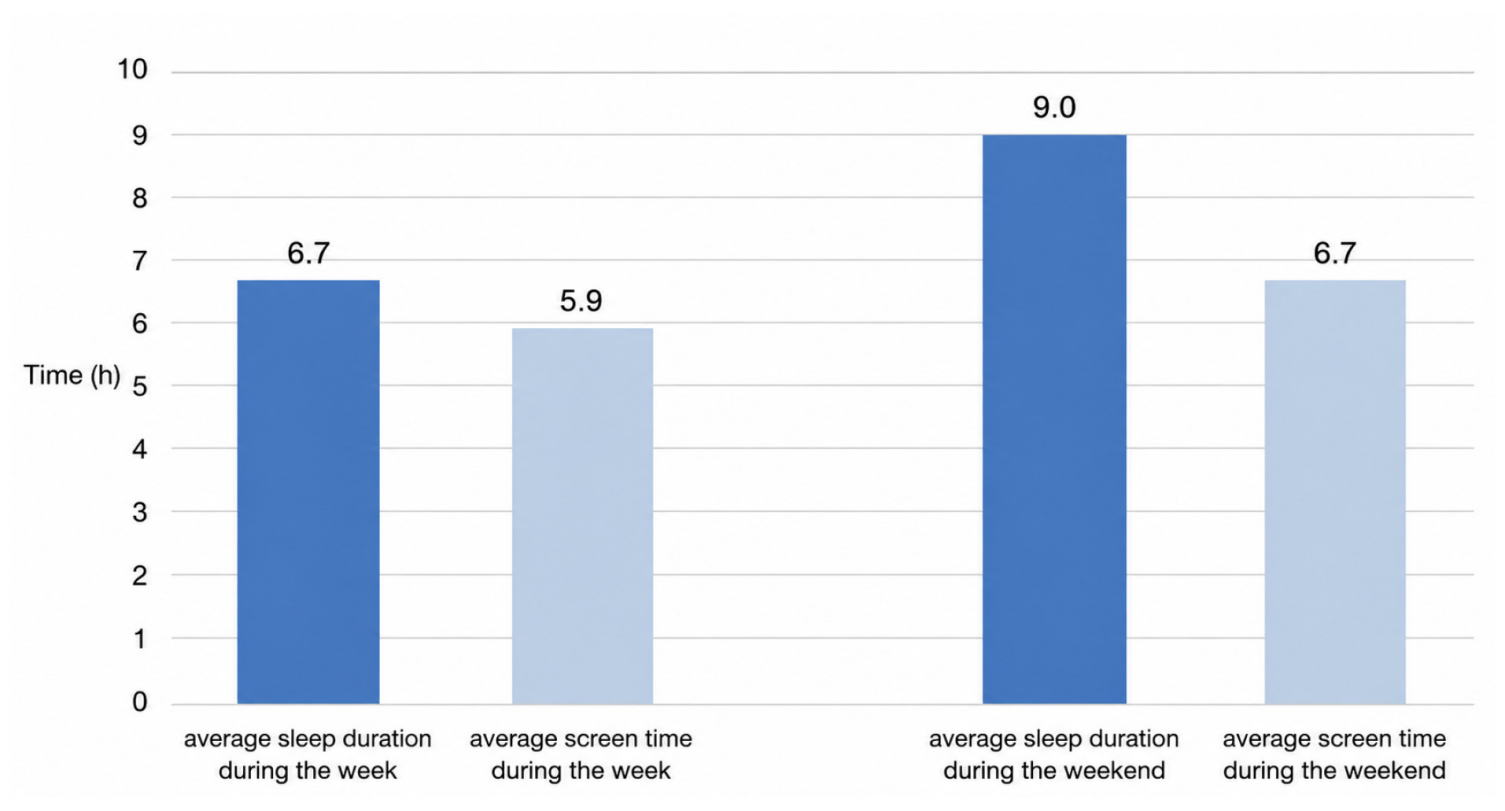
Average sleep time and average screen time during the week and on weekends.

The average sleep duration of respondents was 6.7 ± 1.3 h during the week and increased to 9.0 ± 1.5 h on weekends (*p* < 0.001). During the week, the average time spent in front of a screen by respondents was 5.9 ± 2.6 h and on weekends 6.7 ± 2.8 h (*p* < 0.001) (Figure 1). It is worth noting that as many as 46% of respondents declared that they sleep less than 7 h on weekdays, while almost 95% achieved more than 7 h of sleep on weekends.

People who slept < 7 h spent ≥ 5 h a day in front of a screen more often both during the week (69.3% vs. 63.7%; *p* < 0.001) and on weekends (81.5% vs. 75.8%; *p* < 0.001). Women were more likely than men to exceed 5 h of screen time during the week (69.5% vs. 63.3%; *p* < 0.001), but no differences were observed between women and men on weekends.

The average BMI in both groups was within the normal range (18.5–25 kg/m^2^). For people who slept at least 7 h, the BMI was 22.06 kg/m^2^, while in the group sleeping less than 7 h, it was 21.82 kg/m^2^. When broken down into individual BMI categories, people who slept less than 7 h were significantly more likely to be underweight (BMI < 18.5 kg/m^2^) or obese (BMI ≥ 30 kg/m^2^).

During the summer season, people who slept ≥7 h were more likely to meet the WHO recommendations for vigorous and very vigorous physical activity. There were no significant differences between the groups in meeting the recommendations for moderate physical activity (>150 min./week) and the combined recommendations for moderate or vigorous and very vigorous activity. During the winter season, people who slept ≥7 h significantly more often met the recommendations for both moderate and intense physical activity (Table 1).

**Table 1:** Percentage of respondents declaring compliance with physical activity recommendations in the summer and winter seasons.

Type of activity	< 7 h of sleep	≥7 h of sleep	*p*
Summer			
Moderate >150 min/week	34.6	32.7	0.056
Intense or very intense >75 min/week	56.5	59.3	0.007
Moderate or intense	68.2	69.7	0.11
Winter			
Moderate >150 min/week	17.4	19.8	0.003
Intense or very intense >75 min/week.	35.8	43	< 0.001
Moderate or intense	44.0	51.2	< 0.001

People sleeping < 7 h significantly more often reported consuming any alcohol than people sleeping ≥7 h (53.0% vs. 46.8%, *p* < 0.001), including wine (22.3% vs. 17.2%, *p* < 0.001) and spirits (42.1% vs. 36.2%, *p* < 0.001). The difference in the frequency of alcohol consumption ≥100 g per week was not significant between the groups.

People who slept less reported more frequent current and past tobacco use (60.0% vs. 51.9%; *p* < 0.001) and reported more frequent daily smoking currently (44.1% vs. 36.7%; *p* < 0.001). When analyzing smokers, those who slept less frequently used conventional tobacco products (53.4% vs. 45.3%, *p* < 0.001) and less often smoked only non-classical products (46.2% vs. 54.3% vs. *p* < 0.001).

People who slept < 7 h were significantly more likely to consume a range of highly processed products with high sugar or caffeine content compared to those who slept ≥7 h. A higher percentage of people sleeping < 7 h chose coffee without milk. People sleeping ≥7 h more often consumed red meat and fish. There were no significant differences in the consumption of white meat, vegetables, olive oil, and rapeseed oil (Table 2).

**Table 2:** Frequency of responses to the question about which of the listed products respondents consumed at least once in the last week.

Consumption in the last week	Weekly sleep duration < 7 h (%)	Weekly sleep duration ≥7 h (%)	*p*
Energy drinks	49.6	35.3	< 0.001
Fast food	57.9	54.0	< 0.001
Coffee without milk	33.7	29.7	< 0.001
Red meat (beef, pork)	54.3	57.6	0.003
White meat (chicken, turkey)	85.9	86.8	0.232
Vegetables	93.6	92.7	0.094
Fruit	91.8	90.3	0.019
Olive oil	42.3	41.4	0.442
Rapeseed oil	44.4	46.3	0.088
Chips	50.8	46.9	< 0.001
Sweets	83.4	80.4	< 0.001
Sweetened beverages	70.1	64.7	< 0.001
Fish	39.7	43.7	< 0.001

**Figure 2:**
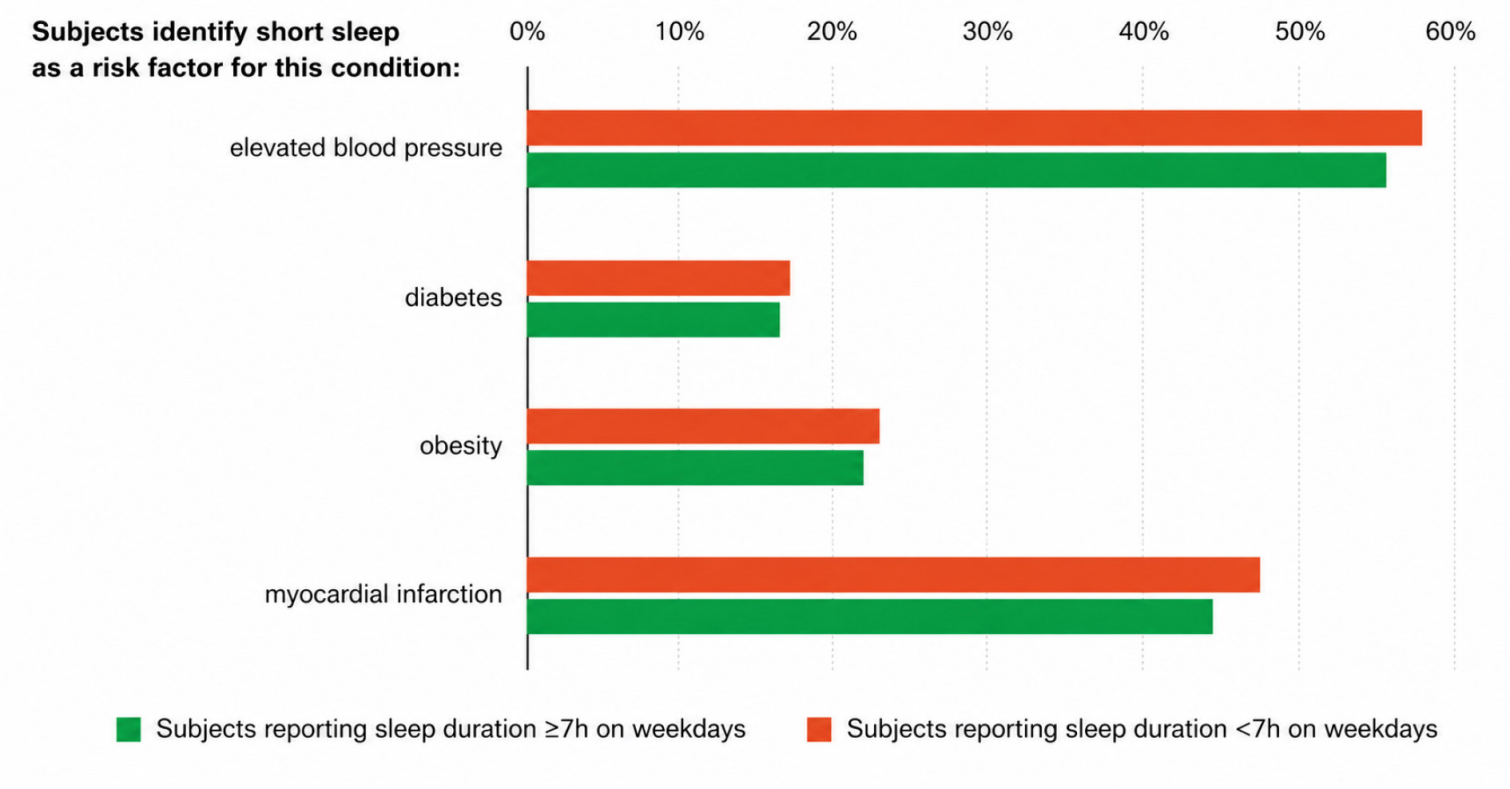
Perception of short sleep as a risk factor for selected diseases among respondents.

In both groups, less than 60% of respondents identified short sleep duration as a factor increasing the risk of high blood pressure, and just under half associated it with the risk of heart attack. Short sleep was much less frequently indicated as a possible cause of obesity—this view was shared by slightly over 20% of respondents, regardless of sleep duration. Respondents most rarely associated sleep deprivation with the risk of developing diabetes (Figure 2).

## Discussion

The findings of our study are concerning, revealing that as many as 46% of final-year secondary school students sleep less than 7 h per night. Our data further demonstrate that short sleep duration is integrally linked to an unhealthy lifestyle, characterized by higher rates of smoking and alcohol consumption, reduced physical activity, and poorer dietary habits. This troubling situation is exacerbated by the low level of awareness among young adults regarding the significant role short sleep time plays in the development of metabolic and cardiovascular diseases.

In our study, almost half of the respondents reported sleeping less than 7 h on weekdays—well below the minimum of 7–9 h recommended by the European Sleep Research Society (ESRS) for young adults. Only 5% continued this deficit on weekends, indicating a widespread attempt to compensate for sleep deprivation—so-called “sleep catching up.” This dramatic discrepancy in sleep time between school days and days off not only exposes a chronic sleep deficit among young people, but also highlights the scale of adaptive behaviors that, although seemingly mitigating the effects, cannot replace a regular, healthy circadian rhythm. Early sleep disturbances in adolescents may be the first step toward the development of CVD in adulthood. This process often begins asymptomatically, and the long-term consequences of reduced sleep may include not only hypertension and obesity, but also lipid disorders, inflammation, and endothelial dysfunction (Martínez-Gómez et al., 2011; De Blas-Zapata et al., 2025).

According to expert opinion from the European Society of Cardiology (ESC), sleep duration and regularity are important modifiable risk factors for cardiovascular disease (CVD). Sleep duration of less than 7 h is considered an independent risk factor for CVD—comparable to hypertension, smoking, and physical inactivity. Moreover, this document highlights that sleep irregularity, regardless of total sleep time, further increases the risk of adverse cardiovascular events (Huang et al., 2025).

This position is based on numerous epidemiological studies and meta-analyses that have shown a significant association between abnormal sleep duration and an increased risk of cardiovascular disease. A large meta-analysis confirmed a U-shaped association between sleep duration and cardiovascular events, with the lowest risk observed at ~7 h of sleep per night. Both short and long sleep were significantly associated with higher risk of coronary heart disease and stroke (Yin et al., 2017).

In our study, we found a significant increase in sleep duration on weekends. The phenomenon of sleep compensation has been well documented in the literature, especially in the context of the mismatch between the circadian rhythm of adolescents and the demands of the school environment (Gradisar et al., 2011). Minges and Redeker (2016) point out that young people are particularly vulnerable to chronic sleep deprivation during the week due to early school start times and educational demands. Additional confirmation of adverse sleep patterns among adolescents comes from the French EnCLASS 2018 study. The average sleep duration on school days among high school students was only 8 h and 14 min, significantly lower than in 2010 (8 h and 35 min). The proportion of students sleeping less than 7 h increased from 25.1% to 29%, and 43.7% reported a sleep deficit exceeding 2 h per day compared with weekends. While comparisons between the EnCLASS cohort and our study population should be interpreted cautiously because of differences in age structure and school characteristics, the findings of both studies point to a common trend of insufficient sleep among young people (Léger et al., 2024). In our study, respondents reported even shorter sleep times on weekdays, averaging 6 h and 40 min. Although longer sleep on weekends may serve a compensatory function, it does not always offset the negative effects of sleep deprivation during the week. Research suggests that such “catch-up sleep” does not fully restore sleep homeostasis and may further disrupt the circadian rhythm, affecting sleepiness during the week and leading to a vicious cycle of sleep deprivation. The latest publication analyzing data from the UK Biobank based on accelerometric sleep measurement did not confirm the beneficial effects of catching up on sleep in reducing the risk of cardiovascular disease (Chaput et al., 2024).

The results of this study confirm the association between reduced sleep duration and longer screen time, both on weekdays and weekends. People who slept less than 7 h per week were significantly more likely to report daily screen time of at least 5 h. An earlier EnCLASS 2018 study also showed a strong influence of screen use in the evening on delayed sleep onset and reduced total sleep time during the working week (Léger et al., 2024). The governments of some European countries (France, Spain, Sweden) are already implementing strategies to reduce excessive screen use by children and adolescents, including bans on mobile phone use in schools and recommendations for no screens in children's bedrooms (Le Monde, 2024; The Guardian, 2024; The Times, 2024).

The average BMI values in both groups analyzed were within the normal weight range. People who slept < 7 h were significantly more likely to be underweight and obese—both extremes are associated with CVD risk (Park et al., 2017). The association between short sleep duration and obesity, considered one of the main modifiable risk factors for cardiovascular disease, has been confirmed in numerous studies. A study by Wang et al. (2023) showed that sleep deprivation during adolescence increases the risk of maintaining abnormal body weight in adulthood, thereby leading to an increased risk of CVD through indirect factors such as hypertension and glycemic disorders. In turn, a systematic review by Sun et al. (2020) indicates that reduced sleep in children and adolescents is significantly associated with higher BMI, blood pressure, and insulin resistance—all of which play a key role in the pathogenesis of cardiovascular disease.

The results of our survey showed that people who slept ≥7 h per day were more likely to meet the WHO recommendations for physical activity, especially in the winter season, indicating that adequate sleep duration may be associated with other health-promoting behaviors. There is growing evidence that shorter sleep duration is associated with lower levels of physical activity among adolescents and young adults. A recent study by Ganz et al. (2022) using data from a large adolescent sample in the United States found a significant positive correlation between sleep duration and physical activity. Adolescents who slept longer reported higher levels of physical activity, even after controlling for sociodemographic factors. These findings suggest that insufficient sleep may contribute to sedentary behavior and that promoting healthy sleep patterns could support physical activity engagement in youth (Ganz et al., 2022). The data obtained indicate that adequate sleep duration may be a factor associated with maintaining adequate physical activity, especially in the winter months when natural activity is reduced.

The results presented in this study indicate that people who slee*p* < 7 h per day are significantly more likely to consume alcohol than those who sleep ≥7 h, including wine and spirits. Although the differences in the reported amount of alcohol consumed (≥100 g/week) did not reach statistical significance, the observed association between shorter sleep and more frequent alcohol consumption remains clinically relevant and consistent with the current state of knowledge. The ERICA study, conducted in Brazil among over 73,000 adolescents aged 12 to 17, identified a frequent co-occurrence of short sleep duration and alcohol consumption, which were among several lifestyle-related cardiovascular risk factors observed particularly in older adolescents (Oliveira et al., 2019). A systematic review of 24 studies confirms that sleep problems correlate with more frequent alcohol consumption among adolescents (Hussain et al., 2020). This relationship is bidirectional: alcohol impairs sleep quality and increases nighttime heart rate, which can burden the cardiovascular system in the long term (De Rosa et al., 2025). A 5-year longitudinal study by Hasler et al. (2022) found that shorter sleep duration and evening chronotype predicted increased alcohol use in adolescents and young adults.

Data from our study show that people who sleep less are more likely to have contact with tobacco, including smoking daily and using traditional tobacco products. A recent study involving over 13,000 U.S. young adults found that both cigarette and e-cigarette use were significantly associated with short sleep duration (< 7 h), with dual users being at the highest risk for inadequate sleep (Merianos et al., 2023). An interesting addition is the observation that people who sleep longer (≥7 h) were more likely to give up traditional tobacco products and were more likely to be former smokers. This may indicate a better overall lifestyle and greater health awareness in this group, which translates into a lower risk of cardiovascular disease.

The results of our survey show that shorter sleep (< 7 h) in young people is associated with more frequent consumption of highly processed foods and beverages containing caffeine and sugar. A recent study by Rocha et al. (2024) analyzing data from over 66,000 Brazilian adolescents found that both short (< 8 h) and long (>10 h) sleep durations were significantly associated with higher intake of ultra-processed foods. Excessive consumption of such foods can lead to obesity, hypertension, and dyslipidemia, which are recognized risk factors for CVD (Dobrowolski et al., 2022). Importantly, people who slept less were more likely to choose energy drinks and coffee without milk—both sources of caffeine, which can disrupt sleep architecture and exacerbate sleep deficit, creating a vicious cycle of circadian rhythm disorders and poor health habits (Young et al., 2020).

Given the high prevalence of short sleep duration among young adults and its well-documented association with unhealthy lifestyles, the findings of our study indicating low awareness of the health consequences of insufficient sleep are particularly concerning. Despite the growing body of evidence supporting the role of short sleep as a risk factor for cardiovascular disease, public awareness in this regard appears to remain limited. Similar observations were reported in a study conducted in India, which showed that most high school students had only average knowledge of cardiovascular risk factors; however, the implementation of an educational program significantly improved their level of awareness (Kumari, 2022).

In summary, these findings underscore the urgent need for targeted public health interventions to improve sleep health and promote healthier lifestyle choices in this vulnerable population, with the potential to significantly reduce future disease burden.

## Data Availability

The raw data supporting the conclusions of this article will be made available by the authors, without undue reservation.

## Supplementary material

Supplementary Figure S1 Graphical summary of weekday and weekend sleep duration, lifestyle factors associated with short sleep, and awareness of short sleep as a cardiovascular and metabolic risk factor among final-year secondary school students.
